# Mentalisation in Parents and Adolescents With Somatic Symptom Disorder: A Cross‐Sectional Case–Control Study

**DOI:** 10.1002/cpp.70327

**Published:** 2026-08-24

**Authors:** Livia Buratta, Gaetano Maria Sciabica, Rosanna Martin, Cristina Venturino, Pasquale Capuozzo, Giulia Ricci, Laura Bandelloni, Marco Crocco, Patrick Luyten, Claudia Mazzeschi, Chiara Pazzagli

**Affiliations:** ^1^ Department of Philosophy, Social Sciences and Education University of Perugia Perugia Italy; ^2^ Department of Dynamic and Clinical Psychology, and Health Studies, Faculty of Medicine and Psychology SAPIENZA University of Rome Rome Italy; ^3^ Paediatric Hospital Psychology Service Azienda Ospedaliera Universitaria Meyer IRCCS Florence Italy; ^4^ Psychology Unit IRCCS Istituto Giannina Gaslini Genoa Italy; ^5^ Pediatric Gastroenterology and Endoscopy Unit IRCCS Istituto Giannina Gaslini Genoa Italy; ^6^ Department of Psychology University of Leuven Leuven Belgium; ^7^ Department of Clinical, Educational and Health Psychology University College London London UK

**Keywords:** emotional awareness, motheradolescence, reflective functioning, somatic symptom disorder

## Abstract

Somatic symptom disorder (SSD) is increasingly prevalent in youth and is associated with impaired later health and social functioning. SSD in youth is linked to emotional awareness and mentalising deficits; however, these factors, together with parental mentalising, have rarely been examined together. This cross‐sectional case–control study aims to investigate these key factors in early adolescents with SSD and their mothers, compared with a community sample. The sample included 360 participants: a clinical group (*n* = 112) of 56 adolescents with SSD (M_age_ = 12.98, SD = 1.72) and their mothers, recruited from two paediatric hospitals; and a comparison group (*n* = 248) of 124 adolescents (M_age_ = 13.04, SD = 1.21) and their mothers from the general population. The adolescents completed the Emotional Awareness Questionnaire (EAQ) and the Reflective Functioning Questionnaire for Youth (RFQ‐Y), and their mothers completed the Parental Reflective Functioning Questionnaire for Adolescents (PRFQ‐A). A logistic regression predicting clinical (vs. comparison) group membership (*R* ^2^ = 0.24; *p* < 0.001) revealed that greater adolescent verbal emotion sharing (OR = 0.41, *p* = 0.015), higher RFQ‐Y curiosity (OR = 0.45, *p* = 0.014) and higher RFQ‐Y certainty (OR = 0.53, *p* = 0.011) were associated with reduced odds of belonging to the clinical group. Additionally, higher maternal PRFQ certainty was linked to lower odds of clinical group membership (OR = 0.56, *p* = 0.003). These findings inform interventions to enhance mentalising and emotional awareness in adolescents with SSD and their mothers, particularly deficits concerning others' mental and affective states.

## Summary

- Clinical assessment of adolescents with somatic symptom disorder should consider emotional awareness and reflective functioning, alongside the evaluation of somatic symptoms.
- Particular attention should be paid to adolescents' difficulties in expressing emotions verbally and in understanding others' mental and emotional states.
- Interventions for somatic symptom disorder may benefit from addressing not only affect labelling but also broader mentalising capacities.
- Assessment and treatment planning should consider parental mentalising, as greater maternal certainty in interpreting adolescents' emotions and thoughts was associated with a lower likelihood of SSD in adolescents.
- Findings support therapeutic approaches that promote the symbolisation and interpersonal processing of emotions underlying somatic symptoms, particularly deficits in emotional investment towards others.

## 1 Introduction

Somatic symptom disorder (SSD) is an increasingly prevalent mental health condition among adolescents, with somatic symptoms affecting approximately 25% of children and adolescents, significantly impacting 10%, and SSD affecting 1%–3% of the population (Díez‐Suárez and Hernández‐González 2025). In paediatric age, SSD is associated with diverse short‐ and long‐term physical and mental health outcomes (Díez‐Suárez and Hernández‐González 2025; Gureje et al. 2001; Rask et al. 2017). Moreover, about 10% of under‐18s consulting paediatricians report disabling yet unexplained symptoms, rendering somatic symptoms a significant public health issue (Konnopka et al. 2012).

To illustrate the clinical burden associated with SSD in adolescence, consider the case of a 13‐year‐old girl brought to the hospital by her parents after months of recurrent abdominal pain and nausea. When asked about her symptoms, she focuses almost exclusively on bodily sensations and struggles to identify or verbalise the emotions associated with her distress, saying only that something ‘feels wrong’ or ‘gets worse when I think about it’. Despite repeated reassurance from healthcare professionals, she remains highly concerned about her physical condition, leading to frequent monitoring of bodily sensations and repeated requests for medical consultations and investigations. She also shows limited curiosity about the emotional or interpersonal factors that might contribute to her symptoms, interpreting her experiences primarily through a physical lens. Over time, her preoccupation with the symptoms intensifies, social interactions with peers diminish, school absences become frequent, and participation in age‐appropriate activities progressively declines.

In adolescents with SSD, common somatic symptoms include abdominal pain, headache, nausea, vomiting, musculoskeletal pain, diarrhoea, dizziness, fatigue, often occurring in the absence of an identifiable medical condition. Their presence may lead to repeated medical consultations and, in many cases, hospital admissions. Recurrent assessments and hospitalisations may further disrupt adolescents' everyday functioning and family life. Studies have shown that SSD in adolescence is often associated with low self‐efficacy and withdrawal behaviours, such as avoiding social activities and school, with significant repercussions for socialisation and academic functioning (van Geelen and Hagquist 2016). Parents may become worried and overwhelmed by the need to provide adequate support for their children's recurring symptoms (Vesterling et al. 2023).

In recent years, clinical understanding of SSD has shifted towards emphasising distress and dysfunction over the presence or absence of physical symptoms. Accordingly, SSD in DSM‐5‐TR (American Psychiatric Association [APA] 2022) is characterised by one or more physical symptoms accompanied by excessive thoughts, feelings, and/or behaviours about the symptoms themselves, resulting in significant distress and/or dysfunction.

Since the pioneering studies on alexithymia (Sifneos et al. 1973), evidence has supported the view that somatic symptoms are associated with difficulties in emotion regulation (De Gucht and Heiser 2003; Kämpfer et al. 2016; Koechlin et al. 2018; Waller and Scheidt 2006). Prerequisites for adaptive emotion regulation in children and adolescents include psychological dimensions, such as emotional awareness and capacities for reflective functioning. Emotional awareness (EA) refers to the degree of attention to and insight into one's own emotional responses and functioning (Rieffe, Terwogt, Petrides, et al. 2007). Previous studies using the Emotion Awareness Questionnaire (Rieffe, Terwogt, Petrides, et al. 2007), a tool developed to examine the role of EA in the aetiology of somatic symptoms, have documented an association between EA and internalising symptoms, including somatic complaints, in adolescence (Barrett et al. 2001; Rieffe, Terwogt, Bosch, et al. 2007; Rieffe et al. 2008; Rieffe and De Rooij 2012; Rueth et al. 2019; Veiga et al. 2019).

In recent years, research has increasingly focused on the role of mentalising in SSD. Mentalising refers to the capacity to interpret self‐ and others' intentional mental states and is a multidimensional construct encompassing implicit and explicit processes, as well as cognitive and affective self–other dimensions; it is considered a prerequisite for effective emotion regulation (Allen et al. 2008, Bateman and Fonagy 2016).

From this perspective, somatic symptoms may partly result from a failure to represent embodied emotional experiences as mental states, a process that may act as both a perpetuating and a predisposing factor in SSD (Ballespí et al. 2019; Bekaroğlu and Yılmaz 2025; Landa et al. 2012; Luyten et al. 2012; Yasky 2025). As outlined by Luyten et al. (2012), somatic symptoms can be understood as a detachment from the ‘lived body’, that is, the body felt from within as the locus of subjectivity, emotion and action in the world. Within an individually shaped vulnerability rooted in biological and environmental factors, the onset of symptoms in response to precipitating events may trigger reliance on strategies aimed at managing escalating stress and anxiety, such as the denial of attachment‐related needs for security and the overemphasis on autonomy. Over time, these strategies may establish a vicious cycle that further impairs mentalising, particularly embodied mentalising. Embodied mentalising refers to the capacity to experience the body as the seat of emotions, desires and feelings and to reflect on one's bodily experiences and sensations and their relation to intentional mental states in self and others (Luyten et al. 2013). Experiences of pain and fatigue progressively erode the capacity to perceive and reflect on bodily signals as representations of inner mental states.

As mentioned above, deficits in mentalising also constitute predisposing factors for the experience of somatic symptoms. From a developmental perspective, caregivers' mentalising capacity plays a fundamental role in children's capacity to represent internal mental states. During early development, caregivers foster children's affective responses through affect sharing and mirroring, which gradually evolve from somatic expression to verbal, playful or imaginative communication of inner emotional states. Caregivers' difficulties with parental mentalising or Parental Reflective Functioning (PRF), namely, the specific capacity to keep their child's mental states in mind, may hinder the development of the capacity for mentalising in children and thus increase the risk for the development of somatic symptoms (Landa et al. 2012; Yasky 2025). The parent–child relationship remains essential during adolescence, and PRF continues to influence adolescents' mentalising (reflective functioning, RF) and overall adjustment (Park and Song 2024).

However, research on RF in early adolescence with SSD remains scarce, despite the high prevalence of somatisation during this developmental stage. Bizzi et al. (2019, 2024) reported significantly lower RF in 8‐ to 15‐year‐olds with SSD compared with healthy controls. Furthermore, Ballespí et al. (2019) found that, among community adolescents, high attention to emotional combined with low emotional understanding was associated with greater somatic complaints, suggesting that detected but poorly mentalised emotions may promote somatisation.

Early adolescence may be a critical developmental period for examining the effects of EA and RF on somatic symptoms, as rapid biological and sociocultural changes occur while mentalising capacities and awareness of one's own feelings are still developing. These developmental demands are thought to underlie the heightened prevalence of somatic symptoms during adolescence (Ballespí et al. 2019; Luyten et al. 2012; Park and Song 2024). Reduced mentalising may impair adolescents' capacity to attribute an appropriate sense to their own and others' emotional experiences and has been associated with poorer emotion regulation, lower resilience and a range of mental disorders (Clarke et al. 2020; Duval et al. 2018; Ha et al. 2013; Lund et al. 2023; Luyten et al. 2020; Martin‐Gagnon et al. 2023; Ortuño‐Sierra et al. 2025).

To our knowledge, no prior study has examined PRF in mothers of young with somatic symptoms, and no research has yet examined EA and RF simultaneously in adolescents with SSD. EA and RF represent related but nonredundant constructs involved in emotional regulation that offer distinct perspectives on symptom development and maintenance. EA refers to the capacity to attend to and differentiate one's own emotions (Rieffe et al. 2008), whereas RF encompasses the broader capacity to interpret self and others' behaviors in terms of intentional mental states across cognitive and affective domains. Examining these constructs jointly may therefore provide a more comprehensive account of the intrapersonal and interpersonal processes underlying SSD.

### 1.1 The Present Study

The present study aims to investigate emotional awareness (EA) and reflective functioning (RF) in early adolescents with SSD, as well as their mothers' parental reflective functioning (PRF), comparing them with community adolescents and their mothers. Emotional awareness and mentalising were assessed using dimensional instruments providing continuous scores across different subscales.

Based on the existing literature, we expected adolescents with SSD to show lower levels of EA and RF than their community peers, as reflected in the scores on subscales indexing poorer emotional awareness and reflective functioning. Similarly, we hypothesised that mothers of adolescents with SSD would show lower levels of PRF, reflected in higher scores on subscales indicating greater difficulties in mentalising their child's mental states. Finally, we explored which adolescent and maternal variables that significantly differed between groups may help explain SSD group membership. Given the exploratory nature of examining these constructs simultaneously, no specific hypotheses regarding their combined effects were formulated. The present study examined parental reflective functioning among mothers of adolescents with SSD and of community adolescents.

## 2 Method

### 2.1 Participants and Procedure

The final sample consisted of 360 participants, including a clinical group (*n* = 112) composed by 56 young adolescents with SSDs (SSD group) and their mothers (*n* = 56), recruited from the Paediatric Hospitals Meyer (Florence, Italy) and Gaslini (Genoa, Italy), after meeting DSM 5 TR diagnostic criteria (APA 2022) during clinical assessment; and a comparison group (CG; *n* = 248) composed by 124 young adolescents, and their mothers (*n* = 124) recruited from the general population. This final sample was obtained after excluding dyads with incomplete questionnaires (SSD group: *n* = 30; CG: *n* = 27) from an initially recruited sample of 237 dyads (474 subjects). The inclusion criteria were as follows: (a) the child was 11 to 17 years old, (b) fluency in Italian, (c) a previous diagnosis of SSD only for the clinical group, and (d) an absence of somatic symptoms for the CG. The mother of the CG group completed the Child Behaviour Checklist 6–18 Version (CBCL; Achenbach and Rescorla 2001) to exclude the presence and intensity of somatic symptoms in their adolescent children. Participation was voluntary, and participants could withdraw at any time. Informed consent was obtained prior to enrollment. The study protocol (269/2020) was approved by the Regional Ethics Committee for Clinical Trials of the Tuscany Region. Data collection took place between 2022 and 2025.

Within the SSD group, hospital psychologists invited mothers and adolescents who met the inclusion criteria to participate in the study. After acceptance and signing the informed consent, the adolescents completed the questionnaire booklet during a clinical interview with the psychologist; at the same time, the mothers completed their questionnaire booklet in a separate room. Within the CG, recruited from the general population, once participation in the study was accepted, both mothers and children completed the questionnaires at home via an online form sent by email. The comparison group was recruited through convenience sampling via personal contacts of the research team and informal word of mouth. This procedure allowed access to a nonclinical comparison sample.

The SSD group of adolescents (*n* = 56) comprised 67.9% females (*n* = 38) with a mean age of 12.98 years (SD = 1.72). The CG adolescents (*n* = 124) comprised 62.1% females (*n* = 77) with a mean age of 13.04 years (SD = 1.21); these distributions did not differ significantly in terms of sex (*χ* ^2^ = 0.555, *p* = 0.456) or age (*U* = 3363, *p* = 0.729). Mothers in the SSD group had a mean age of 47.33 years (SD = 5.09), and mothers in the CG had a mean age of 45.33 years (SD = 4.93), with a significant difference between groups (*U* = 2152, *p* = 0.014). In both groups, most mothers were married or cohabiting, including 83.3% in the SSD group (72.2% married, 11.1% cohabiting) and 65.6% in the CG (55.1% married, 10.5% cohabiting); marital status did not differ significantly between groups (*χ* ^2^ = 4.29, *p* = 0.368). In addition, maternal educational level was assessed (1 = primary/middle school, 2 = high school diploma, 3 = bachelor's or master's degree, 4 = postgraduate qualification/PhD or specialisation). In the SSD group, data on maternal education were available for 53 out of 56 participants. Among these, 15 mothers (28.3%) had a primary/middle school education, 22 (41.5%) had a high school diploma, 16 (30.2%) held a bachelor's or master's degree, and none had a postgraduate qualification. In the CG group (*n* = 124), 15 mothers (12.1%) had a primary/middle school education, 63 (50.8%) had a high school diploma, 32 (25.8%) held a bachelor's or master's degree, and 14 (11.3%) had a postgraduate qualification. A chi‐square test indicated no significant difference in maternal educational level between the SSD and CG groups (*χ* ^2^ = 4.86, *p* = 0.182).

### 2.2 Measures

All measures used in the present study are dimensional instruments that yield continuous scores, with higher values indicating higher levels of the assessed constructs. No validated clinical cut‐offs or categorical classifications are available for these measures. Accordingly, all constructs were interpreted within a dimensional and multidimensional framework consistent with their theoretical conceptualisation, rather than in categorical terms.

#### 2.2.1 Adolescents Self‐Report Questionnaire

Emotional Awareness Questionnaire (EAQ; Rieffe et al. 2008) is a self‐report questionnaire consisting of 30 items. It assesses emotional awareness on a 3‐point Likert scale (1 = *not true*, 2 = *sometimes true*, 3 = *often true*) for use in developmental ages. It assesses six dimensions of emotional awareness: differentiating emotions (DE), referring to the ability to differentiate between emotions and identify their antecedents; verbal sharing of emotions (VS), reflecting the tendency to communicate emotions verbally; not hiding emotions (NE), indicating the tendency not to conceal one's emotions; bodily awareness of emotions (BE), capturing awareness of the bodily symptoms associated with emotional experiences; paying attention to others' emotions (EO), denoting attention to other people's emotional states; and analysis of (one's own) emotions (AE), reflecting the tendency to reflect on and analyse emotions. A higher score represents a higher presence of the corresponding ability, except for Bodily Awareness, in which a higher score implies lower attention to bodily symptoms. The Italian version was used, which showed acceptable to good internal consistency, with Cronbach's alpha coefficients ranging from 0.68 to 0.74 (Camodeca and Rieffe 2013). The present sample showed good internal consistency, with Cronbach's alpha coefficients ranging from 0.70 to 0.81.

Reflective Functioning Questionnaire for Youth (RFQ‐Y; Duval et al. 2018) is a self‐report questionnaire composed of 25 items and rated on a 6‐point Likert scale (1 = *strongly agree*, 6 = *strongly disagree*). It assesses three dimensions of adolescent reflective functioning: Uncertainty/Confusion about mental states (UN), which captures difficulties in mentalising, including confusion, misinterpretation or a lack of clarity about mental states; Certainty about mental states (CMS), which reflects the extent to which adolescents are confident about mental states; and Interest and curiosity in mental states (IC), which indicates openness and interest in exploring inner experiences. The internal consistency values of the original versions were 0.89, 0.80 and 0.75, respectively. For this study, the RFQ‐Y was translated from English into Italian in accordance with the International Test Commission's guidelines for test adaptation (van de Vijver and Hambleton 1996). Two independent forward translations were produced by bilingual translators experienced in translating psychometric instruments. The translators compared their versions and resolved discrepancies through discussion. A committee of clinicians and experts in English and psychology reviewed the integrated version and approved the final version, which pilot testing confirmed was comprehensible to target adolescents. The present sample Italian version showed good internal consistency, with Cronbach's alpha coefficients ranging from 0.71 to 0.85.

#### 2.2.2 Mothers Self‐Report Questionnaire

Parental Reflective Functioning for Adolescents (PRFQ‐A; Luyten et al. 2017a) is a self‐report questionnaire composed of 18 items rated on a 7‐point Likert scale that assesses three different subscales: prementalising modes (PM), reflecting difficulties in understanding the adolescent's mental states; certainty about mental states (CMS), indicating parents' confidence in their interpretations of the adolescent's emotions and thoughts; and interest and curiosity (IC), reflecting the parent's willingness to explore and understand the adolescent's mental states. In the original version, the internal consistency values were 0.62, 0.78 and 0.71 for PM, CMS and IC, respectively. For this study, the PRFQ was also adapted from English into Italian in accordance with the International Test Commission guidelines for test translation (van de Vijver and Hambleton 1996). Two bilingual translators independently produced forward translations of the questionnaire. The translators compared their versions and reconciled discrepancies through discussion. A committee of clinicians and experts in English and psychology reviewed the integrated version and approved the final version, which pilot testing confirmed was comprehensible to target adolescents. In the present sample, the Italian version showed acceptable internal consistency, with Cronbach's alpha coefficients ranging from 0.66 to 0.69.

### 2.3 Data Analysis

Due to the nonnormal distribution of some measures, nonparametric statistics were performed. Descriptive statistics and Mann–Whitney *U* tests were computed to summarise group differences across all studied variables. For all Mann–Whitney *U* tests, the effect size will be reported using the rank‐biserial correlation ($r_{\mathrm{rb}}$), which provides a standardised measure of the magnitude of group differences. A binary logistic regression was run to examine whether individual adolescent and maternal variables that differed significantly between groups were associated with an increased likelihood of belonging to the clinical group (SSD = 1; GC = 0). Findings are reported as odds ratios (ORs) with 95% CI. Statistical significance requires a *p* value < 0.05. All analyses were performed with the software jamovi version 2.7 (The jamovi project 2025) and with the jamovi module GAMLj3 (Gallucci 2019).

## 3 Results

With regard to the first hypothesis, Mann–Whitney *U* tests (Table 1) showed that adolescents in the SSD group reported significantly lower scores in verbal sharing (EAQ‐VS; *p* = 0.013), bodily awareness of emotions (EAQ‐BE; *p* = 0.027), interest/curiosity in mental states (RFQ‐Y IC; *p* < 0.001), and certainty about mental states (RFQ‐Y CMS; *p* < 0.001) compared with adolescents in the CG. No significant differences were found for the other EAQ and RFQ‐Y subscales.

**TABLE 1:** Descriptive statistics and Mann–Whitney *U* test comparisons for all study variables.

Variables	CG M (SD)	SSD M (SD)	*U*	*p*	*r*_*rb*_
EAQ DE	2.23 (0.45)	2.12 (0.47)	2906	0.114	−0.15
EAQ VS	2.00 (0.61)	1.76 (0.62)	2626	0.013	−0.23
EAQ NE	1.94 (0.55)	1.92 (0.60)	3266	0.652	−0.04
EAQ BE	2.00 (0.60)	1.78 (0.60)	2708	0.027	−0.21
EAQ EO	2.65 (0.34)	2.68 (0.36)	3069	0.274	0.10
EAQ AE	2.26 (0.51)	2.0.24 (0.40)	3217	0.543	−0.06
RFQ‐Y UN	3.27 (1.04)	3.06 (0.80)	2927	0.131	−0.14
RFQ‐Y IC	4.35 (0.75)	3.82 (0.69)	2098	< 0.001	−0.38
RFQ‐Y CMS	3.52 (0.98)	2.82 (0.84)	1964	< 0.001	−0.42
PRFQ PM	1.94 (0.84)	2.19 (1.46)	3439	0.920	−0.01
PRFQ CMS	4.31 (1.07)	3.56 (1.00)	2110	< 0.001	−0.39
PRFQ IC	5.52 (1.00)	5.52 (0.99)	3468	0.991	0.00

*Note:* The negative sign indicates lower scores in the SSD group.

Abbreviations: AE = analysis of one's own emotions; BE = bodily awareness of emotions; CMS = certainty about mental states; DE = differentiating emotions; EAQ = emotional awareness questionnaire; EO = paying attention to others' emotions; IC = interest and curiosity; NE = not hiding emotions; PM = prementalising; PRFQ = parental reflective functioning questionnaire; RFQ‐Y = reflective functioning questionnaire for youth; *r*_*rb*_ = rank‐biserial correlation; UN = uncertainty; VS = verbal sharing of emotions.

To address the second aim, group differences in maternal PRF were examined. The Mann–Whitney test showed that mothers in the SSD group reported lower certainty about mental states (PRFQ‐CMS; *p* < 0.001) than mothers in the CG, while differences in prementalising modes and interest/curiosity were not statistically significant.

Finally, we conducted a logistic regression, including the scales that showed significant differences between the two groups (Table 2). Specifically, adolescents' verbal sharing of emotions (EAQ‐VS) and bodily awareness of emotions (BE), two adolescents' RF dimensions, interest/curiosity (RFQ‐Y IC) and certainty about mental states (RFQ‐Y CMS), and maternal certainty about the adolescent's mental states (PRFQ CMS) were entered as predictors of belonging to the SSD group. The overall model was statistically significant (*χ* ^2^ = 52.1; *R* ^2^ = 0.24; *p* < 0.001). Lower EAQ‐VS in adolescents was associated with increased odds of belonging to the clinical group. Similarly, lower adolescent RFQ‐Y IC and lower RFQ‐Y CMS predict an increased risk of belonging to the SSD group. Regarding mothers' parental reflective functioning, lower PRFQ‐CMS was associated with an increased likelihood of being in the SSD group. Finally, levels of bodily awareness of emotions (BE) were not associated with group belonging.

**TABLE 2:** Logistic regression model of psychological adolescents and mothers' dimensions in the SSD group versus the CG.

Variables				95% confidence intervals			
	B	SE	OR	Lower	Upper	*z*	*p*
Intercept	−1.151	0.212	0.316	0.209	0.479	−5.44	< 0.001
EAQ VS	−0.881	0.363	0.414	0.203	0.844	−2.43	0.015
EAQ BE	−0.510	0.354	0.601	0.300	1.202	−1.44	0.150
RFQ‐Y IC	−0.795	0.322	0.452	0.240	0.850	−2.47	0.014
RFQ‐Y CMS	−0.636	0.249	0.530	0.325	0.863	−2.55	0.011
PRFQ CMS	−0.579	0.194	0.560	0.383	0.819	−2.99	0.003

Abbreviations: BE = bodily awareness of emotions; CMS = certainty about mental states; EAQ = emotional awareness questionnaire; IC = interest and curiosity; PRFQ = parental reflective functioning questionnaire; RFQ‐Y = reflective functioning questionnaire for youth; VS = verbal sharing of emotions.

## 4 Discussion

This cross‐sectional case–control study offers an initial contribution to understanding the roles of emotional awareness and reflective functioning in SSD during adolescence. Compared with community peers, adolescents with SSD exhibited greater difficulties in verbally communicating emotions, lower bodily emotional awareness and reduced certainty and curiosity about mental states, while their mothers reported lower confidence in interpreting their children's mental states. Adolescents' emotional awareness and reflective functioning emerged as the strongest correlates of SSD, whereas maternal parental reflective functioning provided an additional, complementary contribution. Overall, our findings indicate that adolescents' difficulties in verbally sharing emotional experiences, reduced curiosity about others' mental states and poorer understanding of them, and mothers' lower confidence in interpreting and appraising their adolescents' emotions and thoughts collectively increase the risk of SSD.

The association between reduced bodily emotional awareness and emotional verbal sharing in adolescents with SSD is consistent with previous evidence (Lahaye et al. 2013; Rieffe, Terwogt, Petrides, et al. 2007; van der Veek et al. 2012; Villanueva Badenes et al. 2016). Rieffe and De Rooij (2012) found that low verbal sharing (e.g., ‘I can easily explain to a friend how I feel inside’), the strongest correlate of SSD membership in our sample, contributed to the prediction of various internalising symptoms, such as depression and fear, by favouring self‐oriented over adaptive external‐oriented attention. Interestingly, this association has not been observed in adults (Villanueva Badenes et al. 2016), suggesting that verbal sharing may represent a particularly salient developmental task during early adolescence, when increased self‐focus and identity formation require more sophisticated capacities for communication and emotion regulation.

Adolescents with SSD also showed poorer reflective functioning, suggesting reduced confidence in understanding others' mental states and a diminished motivation to explore them. This pattern is consistent with previous evidence of impairments in social cognition in individuals with SSDs, including reduced emotional investment in relationships and difficulties in understanding others' mental and affective states (Koelen et al. 2014).

These findings should be interpreted considering the overall RFQ‐Y profile, as the instrument captures complementary dimensions of reflective functioning that are more informative when considered together (Martin‐Gagnon et al. 2023). Contrary to expectations, adolescents with SSD did not report greater uncertainty/confusion about mental states (U/C) than their community peers. Although prior studies have linked U/C with mentalising failure and emotional dysregulation, evidence for its association with SSD and internalising symptoms remains mixed or indirect (Duval et al. 2018; Locati et al. 2023; Parolin et al. 2024).

To clarify these findings, it is helpful to address the self‐other polarities of the construct of mentalising (Ballespí et al. 2021; Luyten et al. 2020). The U/C subscale captures self‐focused thoughts and feelings with a strong affective and behavioural component, whereas CSM primarily assesses cognitive certainty about others' thoughts and feelings (Lund et al. 2023), and IC mainly captures interest and curiosity about others' minds (Park and Song 2024). Building on these distinctions, adolescents with SSD in our sample appeared to exhibit reduced other‐oriented mentalising than their community peers, as reflected in lower IC and CSM scores.

The self–other polarity imbalance is partially consistent with prior research on RF in children with SSD (Bizzi et al. 2019). Yet, unlike this study, our results suggest interpersonal rather than intrapersonal mentalising difficulties. This discrepancy may reflect, at least in part, differences in assessment methods, as self‐report and interview‐based approaches appear to capture only partially overlapping aspects of reflective functioning (Osterhaus and Bosacki 2022; Quesque and Rossetti 2020). Supporting this possibility, Aitken et al. (2026) reported differences in the self–other polarity captured by task‐based and self‐report measures of youth mentalising. Speculatively, high U/C scores, even on self‐reports, are interpreted as reflecting arousal that disrupts mentalising and prompts dysregulated behaviour (Allen et al. 2008). It would be valuable to examine whether adolescents with SSD exhibit lower emotional activation on self‐reports compared with performance‐based measures and relative to peers with other clinical disorders, given their proclivity to somatise emotions.

In relation to parental reflective functioning, lower maternal certainty about mental states (CMS) emerged as a correlate of greater SSD risk in adolescents. While the literature suggests that both excessively low and excessively high certainty may reflect maladaptive forms of parental mentalising—respectively characterised by uncertainty about the child's internal world and overconfidence that overlooks the opacity of mental states (Álvarez et al. 2022; Luyten et al. 2017a, 2017b; Park and Song 2024)—only lower certainty was associated with SSD in the present sample. Recent studies have suggested that the CSM subscale follows a curvilinear pattern (Anis et al. 2020; Stuart et al. 2025), with scores around 4 reflecting optimal parental certainty, whereas both lower and higher scores may indicate hypomentalising and hypermentalising, respectively. Within this framework, mothers of adolescents with SSD in our sample appeared to exhibit a tendency towards hypomentalising. This interpretation is consistent with the view that somatic symptoms may arise, at least in part, from caregivers' difficulties in understanding and making sense of emotional states (Ballespí et al. 2019). Notably, the observed pattern points to difficulties in accurately comprehending their adolescents' internal experiences rather than to reduced interest in or rejection of those experiences, as no group differences emerged for the interest and curiosity (IC) or prementalising (PM) dimensions.

Taken together, these findings suggest that emotional awareness and reflective functioning capture complementary aspects of socio‐emotional functioning in adolescents with SSD. While emotional awareness deficits were primarily related to adolescents' own emotional experiences, as reflected in reduced bodily emotional awareness and greater difficulty verbally sharing emotions, reflective functioning deficits extended to the interpersonal domain, with adolescents showing reduced certainty and curiosity about others' mental states. This pattern suggests difficulties not only in recognising and communicating emotions but also in understanding and anticipating others' thoughts, emotions and intentions. Finally, mothers' lower confidence in interpreting and appraising their adolescents' mental states may further compromise these developmental processes. Difficulties in parental mentalising may reduce caregivers' capacity to accurately recognise and differentiate their children's emotional experiences, thereby limiting opportunities for co‐regulation and reflective dialogue and fostering a greater reliance on somatic expressions of distress, a mechanism recently summarised by Yasky (2025).

The study advances the understanding of SSD by pioneering the exploration of EA and RF using age‐appropriate tools and by assessing both mothers and adolescents. However, the findings should be interpreted with caution due to several limitations. First, the assessment was limited to one parent per adolescent, preventing evaluation of collective reflective functioning in multicaregiver families. Moreover, the predominance of mothers limits generalisability to fathers, given evidence of differential associations between maternal and paternal mentalising and children's socioemotional outcomes (Decarli et al. 2023; Luyten et al. 2017b; Pazzagli et al. 2018). Indeed, in the present study, reflective functioning was assessed exclusively in mothers, as recruitment of fathers proved particularly challenging in both the SSD and control groups. Consequently, fathers' parental reflective functioning was not included, limiting the generalisability of the findings to the broader caregiving context and preventing conclusions regarding potential differences between mothers and fathers. Future studies should include both parents to provide a more comprehensive understanding of parental reflective functioning within the family system.

Second, reliance on self‐report measures may have introduced social desirability and defensive biases, which are partially addressed here through hypotheses aligned with defensive profiles typical of individuals with somatic symptoms. Future research should further clarify differences across assessment methods and compare mentalising profiles across clinical groups. Third, the cross‐sectional design precludes causal inferences, underscoring the need for longitudinal studies to clarify temporal dynamics among variables. Fourth, the use of convenience sampling for the comparison group may have introduced selection bias and limited representativeness. In addition, limitations concern the different modes of administration across groups (in‐person administration by a psychologist in the clinical group vs. online completion in the comparison group), which may have influenced self‐report measures of emotional awareness and reflective functioning due to contextual effects such as social desirability or emotional activation. Furthermore, the absence of broader socioeconomic indicators is acknowledged as a limitation of the present study, as it may limit the full assessment of group comparability. Finally, although the study focused on EA, RF and PRF, future research should consider additional contributors such as adverse childhood experiences, which have been linked to chronic unexplained pain in adulthood (Groenewald et al. 2020).

## 5 Conclusion

This study contributes to theoretical models linking somatic symptoms to adolescent difficulties in affect awareness and mentalising, and maternal challenges in mentalising adolescents' mental states (Landa et al. 2012; Luyten et al. 2012; Yasky 2025). A deeper grasp of the multifactorial contributors to adolescent SSD is essential to inform effective interventions, given the critical role of this developmental period in the onset of chronic and recurrent somatic symptoms.

This is one of the first studies to find an association between other‐mentalising difficulties and SSD, consistent with descriptions of patients with somatic symptoms exhibiting little interest or curiosity about others' mental states. The finding aligns with reports that such patients struggle to cooperate in healthcare settings, form therapeutic alliances in clinical practice, and, more generally, to trust others (Erkic et al. 2018; Kocsis‐Bogar et al. 2026; Luyten et al. 2013).

In the therapeutic treatment of adolescents with SSD, it is essential to attend to difficulties in emotional investment within relationships and in understanding the mental states of others. From a psychodynamic and mentalisation‐based perspective (Luyten et al. 2013), this underscores the importance of prioritising the establishment of a secure therapeutic alliance as a foundational process, within which adolescents can gradually develop greater complexity in their representations of self and others. Central to this process is the clinician's capacity to maintain a reflective stance and to accurately mentalise the adolescent's internal experience, particularly in moments of emotional dysregulation or somatic expression. A key clinical implication of the present study is the need to move beyond interventions focused primarily on emotion labelling in both adolescents and parents. Consistent with evidence questioning the efficacy of standard cognitive‐behavioural approaches in SSD (Erkic et al. 2018; Kleinstäuber et al. 2016), these findings highlight the relevance of targeting deeper processes of symbolisation and mental representation. Treatments should prioritise supporting the symbolisation process of emotions underlying somatic symptoms, particularly deficits in emotional investment towards others. This may be accomplished through therapeutic interventions that focus on the rooting of an individual's internal world in interpersonal relationships, thereby embedding the adolescent's affective life within a relational framework.

## Author Contributions

**Livia Buratta:** data curation, formal analysis, investigation, software, supervision, validation, visualisation, writing – original draft, writing – review and editing. **Gaetano Maria Sciabica:** data curation, formal analysis, visualisation, validation, writing – review and editing. **Rosanna Martin:** conceptualisation, investigation, methodology, project administration, resources, supervision, writing – review and editing. **Cristina Venturino:** conceptualisation, investigation, methodology, project administration, resources, supervision, writing – review and editing. **Pasquale Capuozzo:** data curation, investigation, resources, software, writing – review and editing. **Giulia Ricci:** data curation, investigation, resources, software, writing – review and editing. **Laura Bandelloni:** investigation, resources, supervision, writing – review and editing. **Marco Crocco:** investigation, resources, writing – review and editing. **Patrick Luyten:** supervision, writing – review and editing. **Claudia Mazzeschi:** conceptualisation, methodology, project administration, writing – review and editing. **Chiara Pazzagli:** conceptualisation, funding acquisition, methodology, project administration, resources, supervision, writing – original draft, writing – review and editing.

## Funding

This study was partially funded by Sapienza University of Rome as part of the project ‘Aspects of Psychological Functioning in Parents and Children with Somatic Symptoms’ (SEED) (grant number: 000146_22_BANDO_SEED).

## Conflicts of Interest

The authors declare no conflicts of interest.

## Data Availability

The data that support the findings of this study are available from the corresponding author upon reasonable request.
